# Primary blastic plasmacytoid dendritic cell neoplasm: a US population-based study

**DOI:** 10.3389/fonc.2023.1178147

**Published:** 2023-05-12

**Authors:** Lifang Huang, Fan Wang

**Affiliations:** Department of Hematology, Tongji Hospital, Tongji Medical College, Huazhong University of Science and Technology, Wuhan, Hubei, China

**Keywords:** BPDCN, SEER, survival, age, AFT

## Abstract

**Background:**

Blastic plasmacytoid dendritic cell neoplasm (BPDCN) is a rare and poorly understood hematopoietic malignancy. This study aimed to investigate the clinical characteristics and prognostic factors in patients with primary BPDCN.

**Methods:**

Patients diagnosed with primary BPDCN from 2001 to 2019 were extracted from the Surveillance, Epidemiology and End Results (SEER) database. Survival outcome was analysed with Kaplan-Meier method. Prognostic factors were evaluated based on the univariate and multivariate accelerated failure time (AFT) regression analysis.

**Results:**

A total of 340 primary BPDCN patients were included in this study. The average age was 53.7 ± 19.4 years, with 71.5% being male. The mostly affected sites were lymph nodes (31.8%). Most patients (82.1%) received chemotherapy, while 14.7% received radiation therapy. For all the patients, the 1-year, 3-year, 5-year, and 10-year overall survival (OS) were 68.7%, 49.8%, 43.9%, and 39.2%, respectively, and the corresponding disease-specific survival (DSS) were 73.6%, 56.0%, 50.2%, and 48.1%, respectively. Univariate AFT analysis showed that older age, marital status of divorced, widowed and separated at diagnosis, primary BPDCN only, treatment delay for 3-6 months and without radiation therapy were significantly associated with poor prognosis of primary BPDCN patients. But multivariate AFT analysis indicated that older age was independently associated with worse survival, while second primary malignancies (SPMs) and radiation therapy were independently associated with extended survival.

**Conclusions:**

Primary BPDCN is a rare disease with poor prognosis. Advanced age was linked independently to poorer survival, while SPMs and radiation therapy were linked independently to prolonged survival.

## Introduction

Blastic plasmacytoid dendritic cell neoplasm (BPDCN) is a rare hematopoietic malignancy characterized by aggressive proliferation of precursor plasmacytoid dendritic cells (1), which was firstly identified by Chaperot et al. in 2001 (2). It is estimated that BPDCN may represent 0.44% of all hematologic malignancies (3), and account for 1.8% of all new diagnoses of acute myeloid leukemia (AML) (4). BPDCN occurs in all races and geographic areas (5). It affects all age groups, but most patients are adults, especially older adults, with a median age at diagnosis of 65 to 67 years (5, 6). BPDCN shows a moderate male predominance with an approximate male-to-female ratio of 2.5:1 (7, 8). The most common presentation of BPDCN is asymptomatic skin lesions such as cutaneous nodules and ‘bruise‐like’ patches (7), other sites including lymph nodes, bone marrow (BM) and central nervous system (CNS) are also involved often due to systemic dissemination (9–12). The diagnosis of BPDCN needs the presence of at least 4 of the following 6 antigens: CD4, CD56, CD303/BDCA-2, TCL-1, CD2AP and CD123, along with the absence of lineage-specific markers according to the 5th edition of the World Health Organization Classification of Haematolymphoid Tumours (13). Optimal treatment for BPDCN has not been well defined (14). Traditional chemotherapy regimens modeled after initial induction therapies for acute leukemia and lymphoma can achieve a complete remission (CR) rate of 70% to 90% (6). However, the remissions are mostly short, giving rise to a median overall survival (OS) of approximate 8 to 12 months (15). A retrospective research showed that hyperfractionated cyclophosphamide, vincristine, adriamycin, and dexamethasone (Hyper-CVAD)-based regimens yielded higher complete remission (80% vs 59% vs 43%; P = 0.01), but there was no significant difference in OS compared with other regimens (16). Studies indicate that BPDCN patients who received allogeneic hematopoietic stem cell transplantation (allo-HSCT) during first complete remission had better overall survival compared with those who received chemotherapy alone (median OS, 22.7 months vs 7.1 months) (17, 18). Up to date, BPDCN still remains poorly understood owing to its rarity. In this study, we aimed to delineate the factors affecting the survival of primary BPDCN patients based on a population-based study using the National Cancer Institute’s Surveillance, Epidemiology and End Results (SEER) database.

## Materials and methods

### Data collection

**Figure 1:**
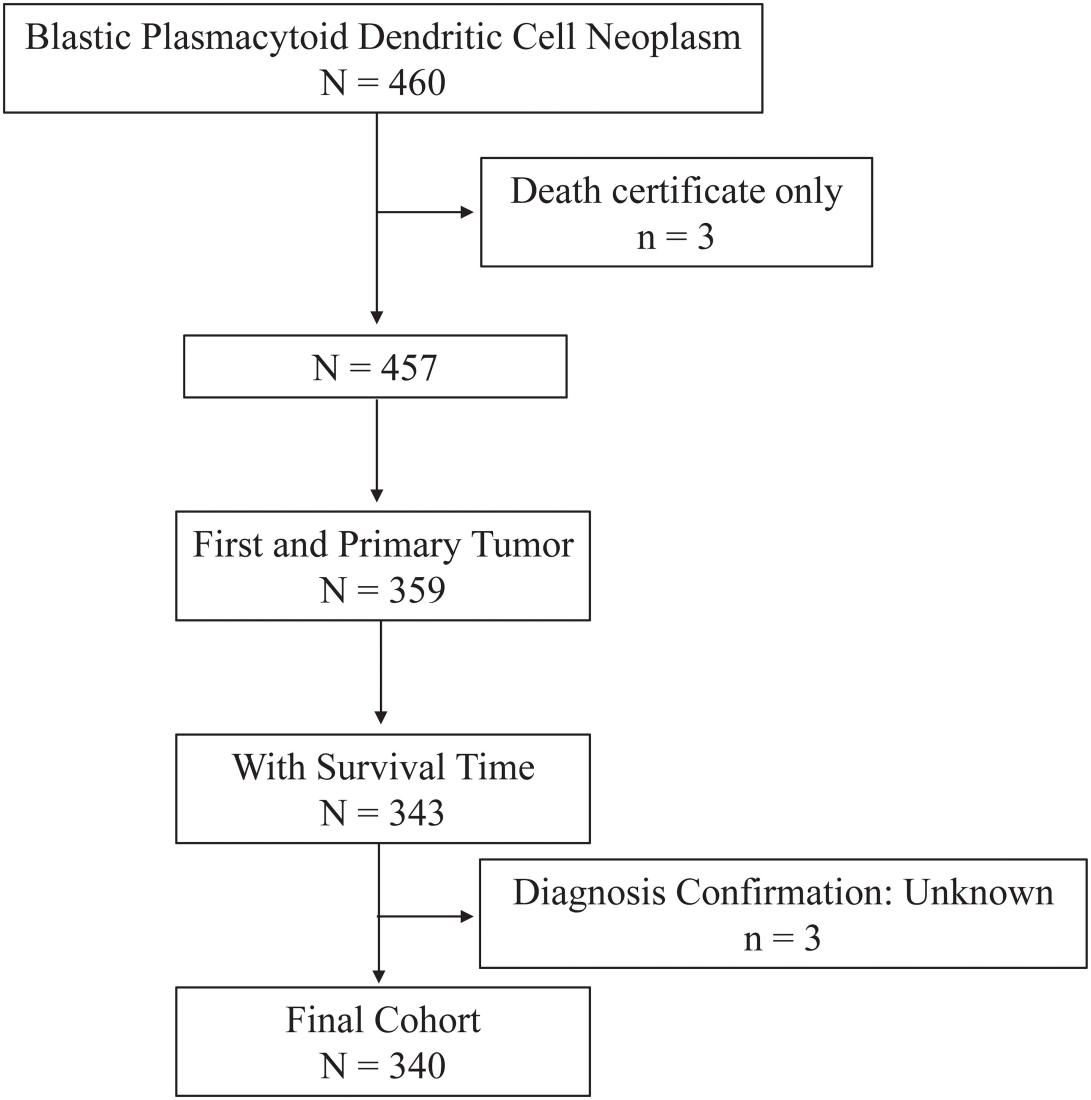
Flow chart of study cohort selection using the SEER database. A flow diagram of BPDCN patient selection in this study. BPDCN, blastic plasmacytoid dendritic cell neoplasm; SEER, Surveillance, Epidemiology, and End Results.

Data for the current study were collected from the Surveillance, Epidemiology, and End Results (SEER) Program (https://seer.cancer.gov/) maintained by the National Cancer Institute (NCI). The SEER*Stat software version 8.4.0.1 (https://seer.cancer.gov/seerstat/, accessed on November 2, 2022) was used to retrieve the data. Through the “Incidence-SEER Research Plus Data, 17 Registries, Nov 2021 Sub (2000-2019),” patients diagnosed with BPDCN between 2001 and 2019 were selected using the case listing session and cases with only known age (censored at age 20-89 years) and only malignant behavior were considered for this study. As shown in the flow chart (**Figure 1**), the inclusion criteria of BPDCN patients were as follows: (1) the International Classification of Diseases for Oncology (ICD-O-3) histologic code (9727/3); (2) the sequence number indicating the only primary or first primary; (3) the patient’s survival time was not 0 or unknown. The exclusion criteria were as follows: (1) the type of reporting source was “death certificate only”; (2) the diagnosis confirmation was unknown. A total of 340 patients with primary BPDCN were included in the final cohort. Since the SEER data used in this study is publicly available and the patient’s private information was anonymous and cannot be reidentified, the ethical approval from the ethics committee was not required.

### Variable definition

The variables collected for analysis included age, gender, race, marital status, year of diagnosis, primary site, vital status, survival months, COD to site recode, cause-specific death classification, cause of death to site, sequence number, first malignant primary indicator, total number of in situ/malignant tumors for patient, type of reporting source, diagnostic confirmation, chemotherapy recode, and radiation recode. Age was defined as the age at diagnosis of BPDCN and were categorized into 4 groups: < 60 years old and 60+ years old. Race were classified into African American, White, and Other (Asian/Pacific Islander, American Indian/Alaska Native, Unknown). Marital status was grouped into married, single, and other (including “Divorced”, “Separated”, “Widowed” and “Unknown”). The variable “COD to site recode,” which was used to analyze the cause of death, was defined based on the International Statistical Classification of Diseases and Related Health Problems, Tenth Revision (ICD-10) codes. The variable “sequence number” was classified into two groups: one primary only and with SPMs (1st of 2 or more primaries, indicating patients had second primary malignancies other than BPDCN). Primary site was grouped into “Skin”, “Bone marrow”, “Lymph nodes” and “Other”. Treatment delay was taken from the variable “Months from diagnosis to treatment”, which measured the interval from diagnosis to the beginning of the initial treatment and was then stratified into four groups: 0, 1-2, 3-6 and Other (including 6 more months and “unknown”). Cause-of-death information was taken from the “COD to site recode” field. BPDCN-related death was defined in the SEER database as “dead (attributed to this cancer diagnosis)”. The definition of BPDCN-unrelated death was defined in the SEER database as death “dead (attributable to causes other than this cancer diagnosis)”. Overall survival time was calculated from the date of BPDCN diagnosis to death or last follow up.

### Statistical analysis

All statistical analysis of the present study was performed using the R program language (http://www.r-project.org/, version 4.2.1; R Foundation for Statistical Computing, Vienna, Austria). Baseline characteristics of primary BPDCN patients were compared between two groups: “alive or BPDCN-unrelated death” and “BPDCN-related death”. Comparisons of continuous data and categorical data were performed using Student’s *t*-test and chi-square test, respectively. The overall survival (OS) and disease-specific survival (DSS) were estimated with the Kaplan-Meier method using the log-rank tests. DSS analysis involves using only deaths identified as being due to BPDCN as the outcome of interest. In the preliminary evaluation of Cox proportional hazards regression by Schoenfeld residual test, it was discovered that several variables violated the proportionality assumption. As a result, an alternative approach was taken using parametric univariate and multivariable accelerated failure time (AFT) regression analysis with the Weibull distribution. All variables that underwent AFT univariate analysis were included in the multivariable AFT model to account for any confounding effects. The resulting coefficients from the AFT analysis were converted into hazard ratios (HR) and 95% confidence intervals (95% CIs) for easier interpretation. All *P* values were two-sided and a *P* value < 0.05 was defined as statistically significant.

## Results

### Baseline characteristics of primary BPDCN patients

As depicted in **Figure 1**, a total 340 patients were finally identified as primary BPDCN in the SEER 17 registry, Nov 2021 Sub (2000-2019) from January 2001 to December 2019. The primary site distribution analysis showed that the mostly affected sites were lymph nodes (*n*=171, 31.8%), bone marrow (*n*=80, 23.5%) and skin (*n*=57, 16.8%). The other sites such as nervous system, testis, lung, spleen, liver, kidney, heart, and colon were rarely affected. A detailed description of primary site distribution of BPDCN was shown in **Supplementary Table S1**. For all patients in the study cohort, 71.5% were male, 2.5 folds that of female (*n*=97, 28.5%; **Table 1**). The estimated average age at diagnosis was 53.7 ± 19.4 years, age distribution was as follows: < 60 years old (53.5%), and 60+ years old (46.5%). The majority of primary BPDCN patients were white (83.2%), African American and Other (including Asian/Pacific Islander, American Indian/Alaska Native and Unknown) occupied 10.6% and 6.2%, respectively. Most of the patients were single who had never been married (59.4%) at diagnosis, followed by married (24.7%) and other marital status (15.9%) including “Divorced”, “Separated”, “Widowed” and “Unknown”. The highest incidence of year range group was 2015- 2019 (30.6%). Most of the patients received treatment in time (41.2%), but 38.2% patients delayed their treatment for 1-2 months, and 5.3% patients delayed the treatment for 3-6 months. For all primary BPDCN cases in this study, 12.1% had second primary malignancies (SPMs). In general, 82.1% primary BPDCN patients received chemotherapy and 14.7% received radiation therapy, respectively. At the time of last follow-up, 150 (44.1%) patients were alive; 153 (45.0%) deaths were attributable to primary BPDCN, and an additional 37 (10.9%) patients died due to other causes such as diseases of heart (n=10, 27.0%), pulmonary disease (n=2, 5.4%), septicemia (n=2, 5.4%), cerebrovascular diseases (n=1, 2.7%), kidney diseases (n=2, 5.4%) and accidents and adverse effects (n=1, 2.7%). Age (*P*= 0.005) and sequence number (*P* = 0.045) were found to be significantly different between the two survival groups “alive or unrelated death” and “BPDCN-related death”. The epidemiologic characteristics and survival comparison were summarized in **Table 1**.

**Table 1:** Comparison of patient baseline characteristics between BPDCN-related death and alive or unrelated death in the primary BPDCN cohort.

Characteristics	Total (N=340)	Alive or Unrelated Death^7^ (N=187)	BPDCN-Related Death^8^ (N=153)	*P*-value
Sex, n (%)				
Male	243 (71.5%)	130 (69.5%)	113 (73.9%)	0.678
Female	97 (28.5%)	57 (30.5%)	40 (26.1%)	
Age, n (%)				
<60	182 (53.5%)	115 (61.5%)	67 (43.8%)	0.005
60+	158 (46.5%)	72 (38.5%)	86 (56.2%)	
Race, n (%)				
White	283 (83.2%)	158 (84.5%)	125 (81.7%)	0.717
African American	36 (10.6%)	16 (8.6%)	20 (13.1%)	
Other^1^	21 (6.2%)	13 (7.0%)	8 (5.2%)	
Marital Status*, n (%)*				
Married	84 (24.7%)	55 (29.4%)	29 (19.0%)	0.127
Single^2^	202 (59.4%)	109 (58.3%)	93 (60.8%)	
Other^3^	54 (15.9%)	23 (12.3%)	31 (20.3%)	
Primary Site*, n (%)*				
Skin	57 (16.8%)	30 (16.0%)	27 (17.6%)	1.000
Bone marrow	80 (23.5%)	45 (24.1%)	35 (22.9%)	
Lymph nodes	171 (50.3%)	95 (50.8%)	76 (49.7%)	
Other^4^	32 (9.4%)	17 (9.1%)	15 (9.8%)	
Diagnosis Year*, n (%)*				
2001-2004	77 (22.6%)	35 (18.7%)	42 (27.5%)	0.306
2005-2009	73 (21.5%)	43 (23.0%)	30 (19.6%)	
2010-2014	86 (25.3%)	43 (23.0%)	43 (28.1%)	
2015-2019	104 (30.6%)	66 (35.3%)	38 (24.8%)	
Sequence Number*, n (%)*				
One primary only	299 (87.9%)	157 (84.0%)	142 (92.8%)	0.045
With SPMs^5^	41 (12.1%)	30 (16.0%)	11 (7.2%)	
Treatment Delay*, n (%)*				
0	140 (41.2%)	78 (41.7%)	62 (40.5%)	0.800
1-2	130 (38.2%)	76 (40.6%)	54 (35.3%)	
3-6	18 (5.3%)	7 (3.7%)	11 (7.2%)	
Other^6^	52 (15.3%)	26 (13.9%)	26 (17.0%)	
Chemo, n (%)				
No/Unknown	61 (17.9%)	31 (16.6%)	30 (19.6%)	0.769
Yes	279 (82.1%)	156 (83.4%)	123 (80.4%)	
RT, n (%)				
No/Unknown	290 (85.3%)	152 (81.3%)	138 (90.2%)	0.070
Yes	50 (14.7%)	35 (18.7%)	15 (9.8%)	

^1^Races including Asian/Pacific Islander, American Indian/Alaska Native and Unknown.

^2^Marital status at diagnosis was single (never married).

^3^Marital status of divorced, widowed and separated at diagnosis.

^4^Primary sites other than”Skin”, “Bone marrow” and “Lymph nodes”.

^5^Sequence number of “1st of 2 or more primaries”, indicating BPDCN patients had second primary malignancies other than BPDCN.

^6^Treatment delay of 6 more months and “unknown”.

^7^Alive or Dead of other cause such as diseases of heart, cerebrovascular diseases, septicemia, and so on.

^8^Death attributable to BPDCN.

BPDCN, blastic plasmacytoid dendritic cell neoplasm; Chemo, chemotherapy; COD, cause of death; RT, radiation therapy; SPMs, second primary malignancies.

Moreover, we analyzed BPDCN as subsequent primary neoplasms (SPNs) from the same cohort by implementing comparable selection criteria to those used for identifying primary BPDCN. Finally, 93 cases were identified as subsequent BPDCN as shown in **Supplementary Table S2**. Interestingly, the estimated mean age of this cohort was 69.2 ± 14.3 years. For all patients in this cohort, most were 60+ years old (75.3%), and 68.8% were male, 2.2 folds that of female (31.2%; **Supplementary Table S2**). The majority of subsequent BPDCN patients were white (91.4%) and most were married (64.5%). Most cases were 2nd of 2 or more primaries (87.1%). The highest diagnosis of year range group was 2015-2019 (46.2%). Most patients received chemotherapy (71.0%). At the time of last follow-up, 19 (20.4%) patients were alive, 74 (79.6%) were dead, and the median survival months was 34.4. Thus, the epidemiologic characteristics of the subsequent BPDCN cohort were quite different from that of primary BPDCN cohort investigated in this study.

### Survival analysis of primary BPDCN patients

**Figure 2:**
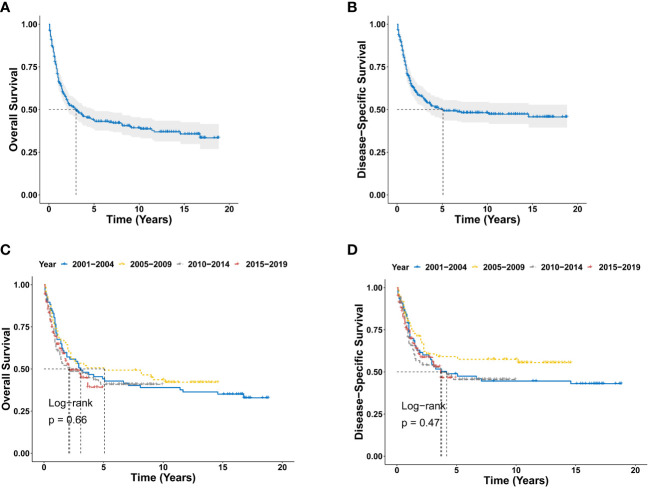
Survival analysis of primary BPDCN. **(A, B)** OS **(A)** and DSS **(B)** curves for all primary BPDCN patients. **(C, D)** Survival curves of OS **(C)** and DSS **(D)** according to the diagnosis years. BPDCN, blastic plasmacytoid dendritic cell neoplasm; OS, overall survival; DSS, disease-specific survival.

**Figure 3:**
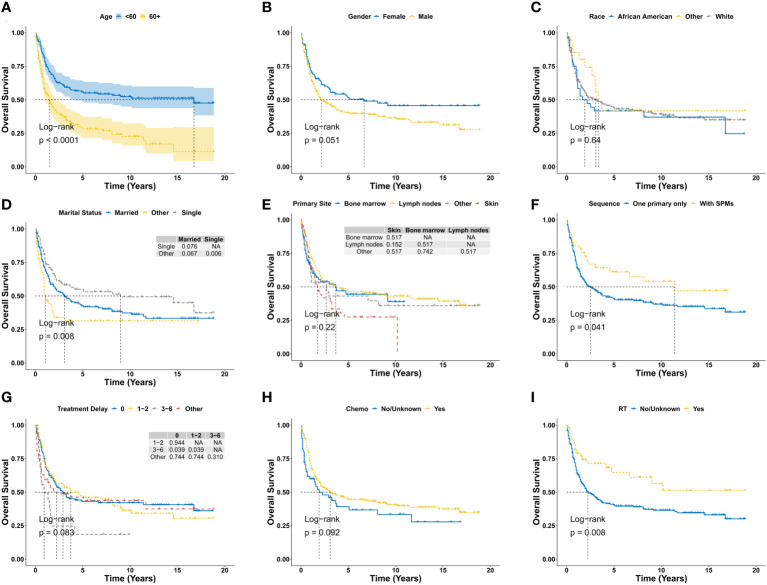
Overall survival analysis of primary BPDCN stratified by age **(A)**, gender **(B)**, race **(C)**, marital status **(D)**, primary site **(E)**, sequence number **(F)**, treatment delay **(G)**, Chemo **(H)** and RT **(I)** using Kaplan-Meier method. BPDCN, blastic plasmacytoid dendritic cell neoplasm; Chemo, chemotherapy; RT, radiation therapy; SPMs, second primary malignancies. NA, Not Available.

**Figure 4:**
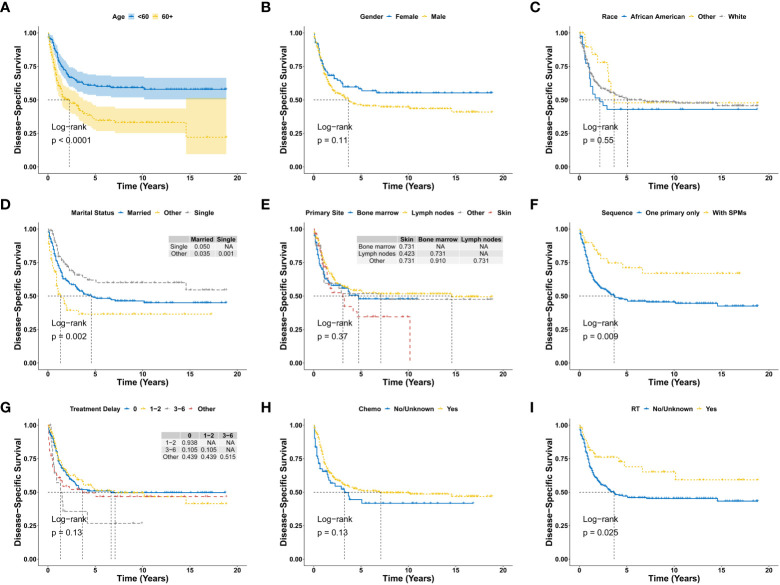
Disease-specific survival analysis of primary BPDCN stratified by age **(A)**, gender **(B)**, race **(C)**, marital status **(D)**, primary site **(E)**, sequence number **(F)**, treatment delay **(G)**, Chemo **(H)** and RT **(I)** using Kaplan-Meier method. BPDCN, blastic plasmacytoid dendritic cell neoplasm; Chemo, chemotherapy; RT, radiation therapy; SPMs, second primary malignancies. NA, Not Available.

There were 190 deaths during the follow-up period, and 153 deaths were disease specific. As shown in **Figures 2A, B**, the 1-year, 3-year, 5-year, and 10-year OS were 68.7%, 49.8%, 43.9%, and 39.2%, respectively, and the corresponding DSS were 73.6%, 56.0%, 50.2%, and 48.1%, respectively. There was no significant difference for OS between the diagnosis year (*P* = 0.66; **Figure 2C**). There were also no significant difference for DSS between the diagnosis years (*P* = 0.47; **Figure 2D**). Furthermore, Kaplan-Meier analysis of OS suggested older age (*P* < 0.0001; **Figure 3A**), marital status of divorced, widowed and separated at diagnosis (*P* = 0.006 vs. single; **Figure 3D**), primary BPDCN only (*P* = 0.041; **Figure 3F**), treatment delay for 3-6 months (*P* = 0.039 vs. “0” and *P* = 0.039 vs. “1-2”; **Figure 3G**), and without radiation therapy (*P* = 0.008; **Figure 3I**) were associated with poorer overall survival. There was no significant difference for overall survival between the variable gender (*P* = 0.051, **Figure 3B**), race categories (*P* = 0.64, **Figure 3C**), primary site (*P* = 0.22, **Figure 3E**) and chemotherapy (*P* = 0.092, **Figure 3H**). Moreover, as shown in **Figure 4**, Kaplan-Meier analysis of DSS showed similar results except for the variable marital status and treatment delay. As shown in **Figure 4D**, the marital status of divorced, widowed and separated showed significantly worse OS (*P* = 0.035 vs. married and *P* = 0.001 vs. single). And there were no significance of DSS for treatment delay (*P* = 0.13, **Figure 4G**).

### Univariate and multivariable AFT regression analysis of primary BPDCN patients

The univariate AFT regression analysis of OS showed that older age, male, marital status of married and other (divorced, widowed and separated) at diagnosis, diagnosis year of 2015-2019, only with primary BPDCN (without SPMs), treatment delay of 3-6 months and without radiation therapy were significantly associated with worse OS (**Table 2**), while the other variables such as, race and chemotherapy did not affect the overall survival outcomes of primary BPDCN. Similar results were obtained with the univariate AFT regression analysis of DSS (**Table 2**). The results of a multivariable AFT regression model that includes all univariate variables were summarized in **Table 3**. Older age (HR: 2.43, CI=1.70-3.49, *P* < 0.001) was found to be associated with worse OS, while with SPMs (HR: 0.56, CI= 0.34-0.92, *P* = 0.022) and radiation therapy (HR: 0.56, CI= 0.34-0.92, *P* = 0.024) was found to be associated with better prognosis. For DSS, older age and marital status of other (divorced, widowed and separated) at diagnosis were significantly associated with worse DSS, and with SPMs was related with better DSS.

**Table 2:** Univariable accelerated failure time regression analysis for OS and DSS of primary BPDCN patients.

Parameters	OS			DSS		
	HR*	95% CI	*P*-value	HR*	95% CI	*P*-value
Gender						
Male	*ref*			*ref*		
Female	0.68	[0.49, 0.94]	0.020	0.70	[0.49, 1.00]	0.049
Age						
<60	*ref*			*ref*		
60+	2.53	[1.89, 3.39]	< 0.001	2.38	[1.72, 3.29]	< 0.001
Race						
White	*ref*			*ref*		
African American	1.08	[0.70, 1.67]	0.739	1.12	[0.73, 1.89]	0.497
Other^1^	0.79	[0.42, 1.50]	0.480	0.80	[0.39, 1.63]	0.534
Marital Status						
Single^2^	*ref*			*ref*		
Married	1.44	[1.01, 2.07]	0.046	1.60	[1.06, 2.43]	0.027
Other^3^	2.21	[1.40, 3.49]	<0.001	2.67	[1.61, 4.45]	< 0.001
Primary Site						
Skin	*ref*			*ref*		
Bone marrow	0.75	[0.47, 1.18]	0.216	0.83	[0.50, 1.37]	0.463
Lymph nodes	0.55	[0.37, 0.83]	0.003	0.56	[0.36, 0.88]	0.463
Other^4^	0.65	[0.37, 1.13]	0.124	4.45	[2.85, 6.94]	0.010
Diagnosis Year						
2001-2004	*ref*			*ref*		
2005-2009	0.94	[0.62, 1.41]	0.766	0.81	[0.51, 1.30]	0.385
2010-2014	1.34	[0.90, 1.98]	0.146	1.37	[0.89, 2.11]	0.143
2015-2019	1.69	[1.12, 2.57]	0.010	1.61	[1.02, 2.55]	0.036
Sequence Number						
One primary only	*ref*			*ref*		
With SPMs^5^	0.57	[0.35, 0.93]	0.024	0.43	[0.23, 0.79]	0.007
Treatment Delay						
0	*ref*			*ref*		
1-2	1.05	[0.76, 1.46]	0.749	1.03	[0.72, 1.49]	0.857
3-6	2.19	[1.24, 3.88]	0.007	2.13	[1.12, 4.06]	0.021
Other^6^	1.10	[0.72, 1.69]	0.652	1.24	[0.79, 1.97]	0.352
Chemo						
No/Unknown	*ref*			*ref*		
Yes	0.73	[0.51, 1.05]	0.090	0.73	[0.49, 1.09]	0.122
RT						
No/Unknown	*ref*			*ref*		
Yes	0.51	[0.31, 0.83]	0.007	0.53	[0.31, 0.91]	0.022

^1^Races including Asian/Pacific Islander, American Indian/Alaska Native and Unknown.

^2^Marital status at diagnosis was single (never married).

^3^Marital status of divorced, widowed and separated at diagnosis.

^4^Primary site other than skin, bone marrow and lymph nodes.

^5^Sequence number of “1st of 2 or more primaries”, indicating BPDCN patients had second primary malignancies other than BPDCN.

^6^Treatment delay of “6+ months” and “Unknown”.

*HR was converted from coefficient in AFT regression model.

BPDCN, blastic plasmacytoid dendritic cell neoplasm; Chemo, chemotherapy; CI, confidence interval; DSS, disease-specific survival; HR, hazard ration; OS, overall survival; RT, radiation therapy; SPMs, second primary malignancies.

**Table 3:** Multivariable accelerated failure time regression analysis for OS and DSS of primary BPDCN patients.

Parameters	OS			DSS		
	HR*	95% CI	*P*-value	HR*	95% CI	*P*-value
Gender						
Male	*ref*			*ref*		
Female	0.75	[0.53, 1.06]	0.108	0.76	[0.51, 1.11]	0.154
Age						
<60	*ref*			*ref*		
60+	2.43	[1.70, 3.49]	< 0.001	2.24	[1.49, 3.35]	< 0.001
Race						
White	*ref*			*ref*		
African American	1.48	[0.93, 2.35]	0.099	1.66	[1.00, 2.74]	0.050
Other^1^	0.94	[0.49, 1.82]	0.855	1.00	[0.48, 2.09]	0.996
Marital Status						
Single^2^	*ref*			*ref*		
Married	1.08	[0.73, 1.60]	0.683	1.28	[0.82, 2.00]	0.276
Other^3^	1.55	[0.95, 2.53]	0.082	1.99	[1.16, 3.44]	0.013
Primary Site						
Skin	*ref*			*ref*		
Bone marrow	0.75	[0.45, 1.23]	0.253	0.83	[0.47, 1.44]	0.496
Lymph nodes	0.79	[0.49, 1.30]	0.360	0.79	[0.46, 1.38]	0.409
Other^4^	1.13	[0.60, 2.13]	0.707	1.06	[0.51, 2.18]	0.874
Diagnosis Year						
2001-2004	*ref*			*ref*		
2005-2009	0.81	[0.52, 1.24]	0.330	0.68	[0.42, 1.11]	0.126
2010-2014	1.10	[0.68, 1.77]	0.697	1.05	[0.62, 1.77]	0.857
2015-2019	1.28	[0.78, 2.11]	0.320	1.15	[0.66, 2.00]	0.621
Sequence Number						
One primary only	*ref*			*ref*		
With SPMs^5^	0.56	[0.34, 0.92]	0.022	0.40	[0.21, 0.76]	0.005
Treatment Delay						
0	*ref*			*ref*		
1-2	0.81	[0.57, 1.16]	0.257	0.84	[0.56, 1.26]	0.404
3-6	0.99	[0.52, 1.88]	0.964	1.00	[0.48, 2.06]	0.996
Other^6^	0.85	[0.49, 1.50]	0.583	1.10	[0.59, 2.03]	0.769
Chemo						
No/Unknown	*ref*			*ref*		
Yes	1.06	[0.63, 1.78]	0.835	1.22	[0.68, 2.20]	0.510
RT						
No/Unknown	*ref*			*ref*		
Yes	0.56	[0.34, 0.92]	0.024	0.59	[0.34, 1.03]	0.062

^1^Races including Asian/Pacific Islander, American Indian/Alaska Native and Unknown.

^2^Marital status at diagnosis was single (never married).

^3^Marital status of divorced, widowed and separated at diagnosis.

^4^Primary site other than skin, bone marrow and lymph nodes.

^5^Sequence number of “1st of 2 or more primaries”, indicating BPDCN patients had second primary malignancies other than BPDCN.

^6^Treatment delay of “6+ months” and “Unknown”.

*HR was converted from coefficient in AFT regression model.

BPDCN, blastic plasmacytoid dendritic cell neoplasm; Chemo, chemotherapy; CI, confidence interval; DSS, disease-specific survival; HR, hazard ration; OS, overall survival; RT, radiation therapy; SPMs, second primary malignancies.

## Discussion

The current study identified 340 primary BPDCN patients of 2001-2019 from the SEER database, representing the largest cohort describing the clinical characteristics and outcome of patients with primary BPDCN. The average age was 53.7 ± 19.4 years, predominantly affected male (male: female = 2.3), and the mostly affected sites were lymph nodes (54.4%). Skin tropism is usually observed in BPDCN, but when tumor cells were initially detected in the bone marrow and the lymph nodes, the diagnosis of BPDCN was primarily based on the immunophenotype of tumor cells from bone marrow or lymph nodes, instead of skin biopsy. This may explain why skin was not the most common primary site in this study. Although BPDCN can occur in any age, studies indicated that it was most common in the elderly patients, with a median age of 61-67 years (19), which slightly differed from our results. The variable demographic characteristics of primary BPDCN in current study may reflect the different age distribution and the rarity and heterogeneity of BPDCN. Moreover, we analyzed BPDCN as subsequent primary neoplasms (SPNs) from the same cohort, and 93 cases were identified as subsequent BPDCN. It was found that the estimated mean age of this cohort was 69.2 ± 14.3 years. But most of the other research did not distinguish primary BPDCN from subsequent BPDCN, while the current study only focused on primary BPDCN, which may explain the age difference of the current study.

Previous studies have demonstrated that age was significantly correlated with the prognosis of BPDCN (1, 5, 7). In the current study, worse OS and DSS were significantly associated with older age, the 5-year OS and DSS rate remarkably declined with increasing age, especially for patients ≥ 60 years of age, which was consistent with prior studies indicating that pediatric BPDCN patients had more favorable clinical outcomes (15). Further univariate and multivariate AFT analysis confirmed that age was an independent prognostic factor for OS and DSS for primary BPDCN patients, which may be partially associated with the decreased intensity of chemotherapies and inability for allo-HSCT as a result of aging (1, 6, 7).

Marital status has been increasingly recognized as an important prognostic factor for cancer patients (20–22). Our study suggested a 1.55-fold hazard ratio of OS and 1.99-fold hazard ratio of DSS in divorced/widowed/separated primary BPDCN patients compared to single ones after multivariable AFT analysis. The underlying mechanism of shortened survival in divorced/widowed/separated patients is unclear, may be related with socioeconomic and psychological status since those patients may have experienced more strong fluctuations in socioeconomic and emotional changes.

Up to now, there is no consensus on the optimal therapeutic regime for BPDCN (14). It has been suggested that traditional leukemia/lymphoma-based chemotherapy regimens did not prolong the overall survival, resulting in only temporary remission, but allo-HSCT especially that performed during the first CR demonstrated durable remissions, with OS rates reaching 74% to 82% at 3 to 4 years (23, 24; 6, 14, 25). Our study showed that although primary BPDCN patients with chemotherapy showed better survival outcome, but the effect was not significant. However, radiation therapy was remarkably associated with longer OS and DSS compared with no radiation therapy group. Univariate AFT analysis suggested that radiotherapy was significantly associated with better prognosis of primary BPDCN patients. However, multivariate AFT analysis showed that radiotherapy was only significantly associated with OS. Furthermore, in the current study, based on population analysis of primary BPDCN patients, we found that patients diagnosed between 2015 and 2019 had no significant different OS and DSS than those diagnosed between 2001 and 2004, reflecting the development status of the novel therapies. Although there were numerous introductions of novel drugs such as Tagraxofusp and Venetoclax for the treatment of BPDCN, which have shown promising results (26, 27), detailed treatment information including such drugs was not documented in the SEER. Thus, it will be hard to analyze the impact of those agents on the prognosis of primary BPDCN patients.

Survival outcome for cancer patients with SPMs can vary depending on the type of cancer and other factors such as the stage of the cancer, age of the patient, and overall health status. Moreover, the presence of a second primary malignancy can complicate the treatment of the initial cancer, which can also impact survival rates. Some research found that breast cancer (28), multiple myeloma (29) or acute lymphoblastic leukemia (30) showed worse survival outcome whey they develop a second primary malignancy. However, some other studies suggest that patients with certain types of cancer, such as breast cancer, small cell lung cancer (31), may have better survival rates if they develop a second primary malignancy. In this study, it was found that SPMs was an independently better prognosis factor for primary BPDCN patients, which could be due to factors such as increased surveillance and earlier detection, better health condition or a more aggressive therapy approach. However, further more research was needed to clarify related mechanisms.

There are several limitations with this study. Firstly, other potential prognostic factors such as LDH level, carcinogens exposure, family history, alcohol/smoking consumption history, Epstein-Barr virus status were not documented in the SEER registry, which may have profound effect on the outcome of BPDCN patients. Secondly, detailed chemotherapy and radiotherapy regimens were not noted in the SEER database, thus it is impossible for us to the analyze the impact of different treatment regimen on the prognosis of BPDCN patients. Thirdly, this is a retrospective study with unavoidable potential biases such as selection bias, recall bias or misclassification bias. Finally, the nomograms of primary BPDCN were constructed and verified by using the same database, which was not further verified by using another independent dataset. Thus, the results of the current study should be interpreted with caution although our study still provided important insights on BPDCN due to the rarity and lacking large-scale trials of the disease.

## Conclusions

In conclusion, primary BPDCN is a rare disease with poor prognosis. Older age, marital status of divorced, widowed and separated at diagnosis, primary BPDCN only, treatment delay for 3-6 months and without radiation therapy were significantly associated with poor survival outcome of primary BPDCN patients. With multivariate AFT regression analysis, older age was independently with worse survival, while SPMs and radiation therapy were independently with extended survival. To our knowledge, this is the largest population-based cohort investigating the clinical characteristics and survival outcome of patients with primary BPDCN.

## Data availability statement

Publicly available datasets were analyzed in this study. This data can be found here: https://seer.cancer.gov/.

## Author contributions

FW came up with the conception, design. FW & LH came up with data analysis and manuscript preparation. Both authors contributed to the article and approved the submitted version.
