# Supplementary material for: Primary blastic plasmacytoid dendritic cell neoplasm: a US population-based study
Source: Front Oncol. 2023 May 12;13:1178147. doi: 10.3389/fonc.2023.1178147 (PMC10213386; doi:10.3389/fonc.2023.1178147)
Supplement: Supplementary file 1 [file Table_1.docx]

**Table S1** Distribution of the primary sites of blastic plasmacytoid dendritic cell neoplasm

| **Primary Sites** | **Overall (N=340)** |
| --- | --- |
| Bladder, NOS | 1 (0.3%) |
| **Bone marrow** | 80 (23.5%) |
| Colon, NOS | 1 (0.3%) |
| Connective, subcutaneous | 8 (2.4%) |
| Fundus of stomach | 1 (0.3%) |
| Kidney, NOS | 1 (0.3%) |
| Lateral wall of bladder | 1 (0.3%) |
| Liver | 1 (0.3%) |
| Long bones | 2 (0.6%) |
| **Lymph nodes** | 171 (31.8%) |
| Abdominal lymph nodes | 8 (2.4%) |
| Intrathoracic lymph nodes | 30 (8.8%) |
| Lymph node, NOS | 43 (12.6%) |
| Lymph nodes of axilla or arm | 2 (0.6%) |
| Lymph nodes of head, face & neck | 9 (2.6%) |
| Lymph nodes of inguinal region or leg | 2 (0.6%) |
| Lymph nodes of multiple regions | 74 (21.8%) |
| Pelvic lymph nodes | 3 (0.9%) |
| **Mediastinum** | 6 (1.8%) |
| Anterior mediastinum | 4 (1.2%) |
| Mediastinum, NOS | 2 (0.6%) |
| **Nervous system** | 2 (0.6%) |
| Brain, NOS | 1 (0.3%) |
| Overlapping lesion of brain & CNS | 1 (0.3%) |
| Orbit, NOS | 2 (0.4%) |
| Ovary | 1 (0.2%) |
| **Overlapping lesion** | 2 (0.6%) |
| Overlapping lesion of breast | 1 (0.3%) |
| Overlapping lesion of skin | 1 (0.3%) |
| Parotid gland | 1 (0.3%) |
| Pharynx, NOS | 1 (0.3%) |
| **Skin** | 57 (16.8%) |
| Skin of lower limb and hip | 3 (0.9%) |
| Skin of scalp and neck | 2 (0.6%) |
| Skin of trunk | 13 (3.8%) |
| Skin of upper limb and shoulder | 9 (2.6%) |
| Skin other/unspec parts of face | 5 (1.5%) |
| Skin, NOS | 24 (7.1%) |
| Overlapping lesion of skin | 1 (0.3%) |
| Small intestine, NOS | 1 (0.3%) |
| Spleen | 1 (0.3%) |
| Testis | 1 (0.3%) |
| Tonsil | 1 (0.3%) |
| Upper lobe, lung | 1 (0.3%) |

Abbreviations: NOS, not otherwise specified.
