# Supplementary material for: Primary blastic plasmacytoid dendritic cell neoplasm: a US population-based study
Source: Front Oncol. 2023 May 12;13:1178147. doi: 10.3389/fonc.2023.1178147 (PMC10213386; doi:10.3389/fonc.2023.1178147)
Supplement: Supplementary file 2 [file Table_2.docx]

**Table S2** Baseline characteristics of the subsequent BPDCN cohort

| **Characteristics** | **Overall (N=93)** |
| --- | --- |
| **Sex** |  |
| Male | 64 (68.8%) |
| Female | 29 (31.2%) |
| **Age** |  |
| <60 | 23 (24.7%) |
| 60+ | 70 (75.3%) |
| **Race** |  |
| White | 85 (91.4%) |
| African American | 6 (6.5%) |
| Other^1^ | 2 (2.2%) |
| **Marital Status** |  |
| Single^2^ | 8 (8.6%) |
| Married | 60 (64.5%) |
| Other^3^ | 25 (26.9%) |
| **Primary Site** |  |
| Skin | 21 (22.6%) |
| Bone marrow | 27 (29.0%) |
| Lymph nodes | 41 (44.1%) |
| Other^4^ | 4 (4.3%) |
| **Sequence number** |  |
| 2nd of 2 or more primaries | 81 (87.1%) |
| 3rd of 3 or more primaries | 10 (10.8%) |
| 4th of 4 or more primaries | 1 (1.1%) |
| 5th of 5 or more primaries | 1 (1.1%) |
| **Diagnosis Year** |  |
| 2001-2004 | 12 (12.9%) |
| 2005-2009 | 12 (12.9%) |
| 2010-2014 | 26 (28.0%) |
| 2015-2019 | 43 (46.2%) |
| **Treatment Delay** |  |
| 0 | 27 (29.0%) |
| 1-2 | 34 (36.6%) |
| 3-6 | 7 (7.5%) |
| Other^5^ | 25 (26.9%) |
| **Chemo** |  |
| No/Unknown | 27 (29.0%) |
| Yes | 66 (71.0%) |
| **RT** |  |
| No/Unknown | 83 (89.2%) |
| Yes | 10 (10.8%) |
| **Survival Status** |  |
| Alive | 19 (20.4%) |
| Dead | 74 (79.6%) |
| **Survival months** |  |
| Mean (SD) | 34.4 (51.3) |
| Median [Min, Max] | 13.0 [1.00, 217] |

^1^ Races including Asian/Pacific Islander, American Indian/Alaska Native and Unknown.

^2^ Marital status at diagnosis was single (never married).

^3^ Marital status of divorced, widowed and separated at diagnosis.

^4^ Primary sites other than“Skin”, “Bone marrow” and “Lymph nodes”.

^5^ Treatment delay of 6 more months and “unknown”.

Abbreviations: BPDCN, blastic plasmacytoid dendritic cell neoplasm; Chemo, chemotherapy; RT, radiation therapy.
